# Role of Glia-Derived Extracellular Vesicles in Neurodegenerative Diseases

**DOI:** 10.3389/fnagi.2021.765395

**Published:** 2021-10-20

**Authors:** Tianbai Li, Xiang Tan, Song Li, Murad Al-Nusaif, Weidong Le

**Affiliations:** ^1^Liaoning Provincial Key Laboratory for Research on the Pathogenic Mechanisms of Neurological Diseases, The First Affiliated Hospital, Dalian Medical University, Dalian, China; ^2^Institute of Neurology, Sichuan Academy of Medical Sciences, Sichuan Provincial People’s Hospital, Chengdu, China

**Keywords:** extracellular vesicles, glia, neurodegenerative diseases, astrocyte, microglia

## Abstract

Extracellular vesicles (EVs), as nano-sized vesicles secreted by almost all cells, have been recognized as the essential transmitter for cell-to-cell communication and participating in multiple biological processes. Neurodegenerative diseases (ND), such as Alzheimer’s disease, Parkinson’s disease, and amyotrophic lateral sclerosis, share common mechanisms of the aggregation and propagation of distinct pathologic proteins among cells in the nervous systems and neuroinflammatory reactions mediated by glia during the pathogenic process. This feature indicates the vital role of crosstalk between neurons and glia in the pathogenesis of ND. In recent years, glia-derived EVs have been investigated as potential mediators of signals between neurons and glia, which provides a new direction and strategy for understanding ND. By a comprehensive summary, it can be concluded that glia-derived EVs have both a beneficial and/or a detrimental effect in the process of ND. Therefore, this review article conveys the role of glia-derived EVs in the pathogenesis of ND and raises current limitations of their potential application in the diagnosis and treatment of ND.

## Introduction

Neurodegenerative diseases (ND) are a varied assortment of central nervous system (CNS) disorders characterized by the progressive loss of neurons and appearance of abnormal proteinaceous assemblies in the nervous system, such as Alzheimer’s disease (AD), Parkinson’s disease (PD), and amyotrophic lateral sclerosis (ALS) (Rachakonda et al., 2004; Jucker and Walker, 2018; DeTure and Dickson, 2019). Although significant achievements in the knowledge on pathogenic mechanisms of ND have been made, there is still an urgent need and has been actively pursued in the field for reliable biomarkers for early diagnosis and management of the disease course (Li and Le, 2017; Zetterberg and Biennow, 2021). An even more serious concern is that ND, as a major health problem for the world’s aging population, has no cure in sight. To overcome these obstacles, a growing body of research is focusing on the role of cell-to-cell communications in the pathogenesis of ND (Mathieu et al., 2019). It has been shown that glia-neuron crosstalk in the CNS is fundamental for various biological functions, ranging from brain development, neural circuit maturation, and homeostasis maintenance. Understanding how to assess and modulate glia-neuron interactions is essential for developing effective therapies for ND (Bartels et al., 2020).

The crucial role of extracellular vesicles (EVs) in glia-to-neuron communications has been recognized in recent years. EVs are cystic vesicles with a double-layer membrane structure, first identified from the blood (Wolf, 1967). Later studies further discovered that almost all eukaryotic cells could secrete EVs, such as stem cells, various organ cells, and cells in the CNS, including neurons, astrocytes, microglia, and oligodendrocytes (Budnik et al., 2016). EVs can be classified according to cell sources or the origin of their membrane. Using the latter categorization, EVs can be divided into microvesicles (100–1,000 nm in diameter) and exosomes (30–50 nm diameter) (van Niel et al., 2018). The emerging data from electron microscopy, flow cytometry, high-throughput proteomics, and genomics have confirmed that EVs contain plentiful amounts of cell components and can migrate to specific organs or cells, thereby diverting the contents into the recipient to modulate the state of the cell (Vinuesa et al., 2019). The characteristics of EVs determine the functions of glia-derived EVs on the glia-neuron crosstalk, which provides enormous potential for investigating the pathogenesis and new therapeutic targets for ND.

In this review, we will summarize the advances of EV research in the field of ND, with a specific focus on glia-derived EVs. Effects of glia-derived EVs in the inter-cellular communications among astrocytes, microglia, oligodendrocytes, and neurons are described. Furthermore, two distinct roles: beneficial and detrimental roles of glia-derived EVs in the pathogenesis of typical ND, including AD, PD, and ALS, are particularly discussed. We also provide an update on the glia-derived EVs as potential biomarkers for ND. Recent developments on the application of glia-derived EVs as new nanotherapeutics are also briefly reviewed here.

## Extracellular Vesicles in Glia-Neuron Communication

### Astrocyte-Derived Extracellular Vesicles on Neurons

Astrocytes are the most abundant cell type in the brain. They actively contribute to various essential functions in the CNS, including the formation and maintenance of the blood-brain barrier (BBB), synapse formation and plasticity, ion homeostasis, neurotransmitter buffering, and the secretion of neuroactive agents (Freeman, 2010; Linnerbauer et al., 2020). Neurons and astrocytes orchestrate CNS homeostasis through multiple mechanisms of transcellular communication, which range from local cell-to-cell direct contact to the release of neurotransmitters and EVs (Huang et al., 2018).

Constitutively secreted astrocyte-derived EVs have now emerged as crucial players in maintaining normal neuronal functions, including promoting neurite outgrowth and neuronal survival (Chaudhuri et al., 2018). For example, prion protein (PrP) in astrocytes involves the perception of oxidative stress. The release of EVs carrying PrP from astrocytes may improve the survival of neurons under hypoxic and ischemic conditions (Guitart et al., 2016). Apolipoprotein D (ApoD), a classical neuroprotective protein, can be exclusively transported by EVs from astrocytes to neurons, thereby promoting functional integrity and survival of neurons against oxidative stress (Pascua-Maestro et al., 2019). In addition, *Clostridium botulinum* C3 transferase (C3bot) has vimentin-dependent axonotrophic effects and may participate in the molecular crosstalk between astrocytes and neurons. A study presented evidence that the astrocytic release of vimentin by EVs has neuro-regenerative and plasticity augmenting effects through the interaction of C3bot with neuronal membranes after spinal cord injury (Adolf et al., 2019). Besides, there is evidence that astrocytes can regulate the dendritic development of neurons by the cargo miRNA and mtDNA of their derived EVs (Guescini et al., 2010; Luarte et al., 2020).

What’s more, astrocytes can alter the cargo of astrocyte-derived EVs in response to different stimuli, such as neuroinflammation (Chaudhuri et al., 2020; You et al., 2020). Recent evidence showed that human astrocyte-derived EVs activated by interleukin (IL)-1β could reduce the neurite outgrowth, branching, and neuronal firing of the primary cultured mouse cortical neurons (You et al., 2020). Astrocytes-derived EVs secreted in response to IL-1β and tumor necrosis factor (TNF)-α were enriched with miRNAs that target proteins involved in neurotrophin signaling. Downregulation of the target genes of these miRNAs in neurons was associated with reductions in dendritic growth, dendritic complexity, reduced spike rates, and burst activity (Chaudhuri et al., 2020). However, contrary to the above, astrocyte-derived EVs have neuroprotective effects when triggered by certain conditions. It has been shown that astrocytes, when subjected to hyperthermia, can secrete EVs containing heat shock protein Hsp/c70, as well as intracellular signaling components including activated forms of extracellular-signal-regulated kinase, Akt, and Jun N-terminal kinase (JNK)/SAPK that may have implications for the survival of neurons (Taylor et al., 2007). Studies have also demonstrated that astrocytes-derived EVs can inhibit autophagy and ameliorated neuronal damage in oxygen and glucose deprivation conditions by releasing specific microRNAs (Pei et al., 2020; Xu et al., 2019). A quantitative proteomic profile analysis aims to explore the cargo of astrocyte-derived EVs in response to various stimuli and found that EVs from astrocytes treated with ATP and IL-10 contains a series of proteins that play a role in enhancing neurite outgrowth, regulation of synaptic transmission, dendritic branching, and promoting neuronal survival. On the contrary, astrocyte-derived EVs released in response to IL-1β comprise proteins that regulate peripheral inflammatory response and immune cell trafficking to the CNS (Chaudhuri et al., 2018). From the evidence above, it can be concluded that different types of stimuli have opposed effects on the survival of the neurons. The impact degree and mechanisms of these stimuli on EVs releasing remain to be further studied.

### Microglia-Derived Extracellular Vesicles on Neurons

Microglia account for approximately 10% of cells in the CNS, which usually exist in a resting state and contribute to modulating the strength of synaptic transmissions and sculpting neuronal synapses (Colonna and Butovsky, 2017). Microglia appear to be heterogeneous with diverse functional phenotypes that range from pro-inflammatory M1 phenotypes to immunosuppressive M2 phenotypes (Tang and Le, 2016; Zhang et al., 2017; Wolters et al., 2021). Once sensing damage signals or toxic aggregated proteins inside or outside the cell, microglia will switch from resting to active (Zhong et al., 2018; Dong et al., 2020). The continuous crosstalk between microglia and neurons is dependent on microglia housekeeping functions and contributes to the homeostasis of CNS (Marinelli et al., 2019). In addition to the chemokines and cytokines, an increasing body of evidence indicates that EVs are also crucial to microglia-neuron communication.

Microglia-derived EVs have been shown to participate in the miscellaneous physiological functions, including metabolic supporting of neurons, regulating synaptic activity and transmission, as well as neuronal survival. For instance, a proteomic profile of the microglial-derived EVs from primary microglia following treatment with recombinant carrier-free Wnt3a showed that the altered proteins are involved in cellular architecture, metabolism, protein synthesis, and degradation (Hooper et al., 2012). In a lipotoxic context emulated by incubating primary microglia with palmitate, microglial-derived EVs induced the morphologic alterations of the dendritic spine in primary hippocampal neurons (Vinuesa et al., 2019). Concerning the function of neuronal survival, M2 microglia-derived EVs were demonstrated to attenuate ischemic brain injury and promoted neuronal survival via miR-124 and its downstream target USP14, which have been shown to participate in the regulation of brain-derived neurotrophic factor and fibroblast growth factor (Song et al., 2019). As neuroprotective cargos, other miRNAs have also been reported to be altered in the microglial-derived EVs. And they could be the EVs-dependent connections between microglia and neurons, which is worth researching further (Lemaire et al., 2019).

Plenty of evidence indicates that different subsets of microglial-derived EVs have diverse functional properties, and specific signaling pathways may regulate the trafficking of EVs. On the one hand, EVs from pro-inflammatory microglia (M1 phenotype) have been shown to contribute to the pro-neuroinflammation response. EVs contained pro-inflammatory molecules initially released by microglia following the external stimulus can activate more microglia that may contribute to the progressive neuroinflammatory response (Kumar et al., 2017). Besides, the crosstalk between EVs and inflammasome is becoming one of the hotspots in inflammatory reactions (Noonin and Thongboonkerd, 2021). It has been discovered that lipopolysaccharide (LPS)-primed microglia exposed to manganese secrete more EVs that contain ASC, a component of the inflammasome complex. And ASC, in turn, leads to the increase of NLRP3 and pro-IL-1β in microglia, thus promoting the inflammasome activation (Sarkar et al., 2019). What’s more, a distinct profile of proteins was identified in EVs released from LPS treated microglia compared to the control (Yang et al., 2018). The research aims to explore the relationships among inflammatory microglia, their released EVs, and the synaptic defects of neurons, has identified miR-146a-5p, a microglia-specific miRNA, targets two kinds of adhesion protein that play a crucial role in dendritic spine formation and synaptic stability. The results also show that inflammatory EVs transfer miR-146a-5p cargo to neurons and significantly decrease dendritic spine density in hippocampal neurons (Prada et al., 2018).

On the other hand, microglia-derived EVs may also have a protective effect on neurons through the anti-inflammatory response (M2 phenotype). There’s research shows that microglia-derived EVs carrying inflammation-related genes can transfer signals to the glioma cells in the brain, thereby playing a role in reducing neuronal death and promoting the recovery of brain homeostasis (Grimaldi et al., 2019). When cultured with brain extracts of traumatic brain injury mice, EVs released from microglia have a significantly higher level of miR-124-3p, promoting the anti-inflammatory M2 polarization in microglia and ameliorating the inflammatory reaction in scratch-injured neurons (Huang et al., 2018). The above evidence indicates the influence of microglia-derived EVs subjected to an inflammatory environment can be detrimental or beneficial is depended on the context, supporting the need to investigate the involvement of microglial-derived EVs in different pathological conditions in more detail.

### Oligodendrocyte-Derived Extracellular Vesicles on Neurons

The role of oligodendrocytes in the CNS is to insulate axons, maintain axonal health and transmit the rapid impulse with a multilayered myelin sheath. The formation of functional myelin sheath-axon entity depends on the interactions between the oligodendrocyte and neuron. Neurons can internalize oligodendrocyte-derived EVs in response to multiple neuronal signals and present neuroprotective properties in the CNS (Krmer-Albers, 2020). Previous research found that oligodendrocyte-derived EVs carrying specific protein and RNA cargo can be secreted under the stimulation of neurotransmitter glutamate triggers mediated by Ca^2+^ entry through oligodendroglial NMDA and AMPA receptors. Notably, these oligodendrocyte-derived EVs have multiple effects on neurons under conditions of cell stress, including improving neuronal viability, modulating neuronal outgrowth and metabolism (Frühbeis et al., 2013). The same research team went on to show that oligodendrocyte-derived EVs contribute to resisting oxidative stress and promoting neuronal survival through the diversion of superoxide dismutase and catalase during oxygen-glucose deprivation (Fröhlich et al., 2014). Analyses of two mouse models deficient in oligodendrocyte-specific genes showed that wild-type oligodendrocyte-derived EVs improve the metabolic state and promote axonal transport in nutrient-deprived neurons. In contrast to that, mutant oligodendrocytes release fewer EVs along with underrepresented proteins cargo. Notably, mutant oligodendrocyte-derived EVs lost the function of maintaining the nutrient-deprived neurons and promoting axonal transport (Frühbeis et al., 2020).

## Extracellular Vesicles in Glia-To-Glia Communication

In addition to acting on neurons, recent studies have shown that glia-derived EVs also play a vital role in intercellular glia-to-glia communications. It has been demonstrated that EVs secreted from young astrocytes, but not those from aged astrocytes, can convey support for the differentiation of oligodendrocytes through altering the expression of proteins in oligodendrocyte progenitor cells, resulting in the maturation and survival of oligodendrocyte (Willis et al., 2020). A recent study discovered that EVs derived from activated astrocytes could accelerate the transformation of the microglial anti-inflammatory phenotype. And miR-873a-5p, as one of the components of these astrocyte-derived EVs, may play a vital role in the interactions between astrocytes and microglia (Long et al., 2020). There is also evidence that EVs released by pro-inflammatory microglia can block remyelination.

In contrast, EVs from microglia and mesenchymal stem cells co-cultured systems contribute to the recruitment of oligodendrocyte precursor cells (OPC) and myelin repair. Moreover, astrocytes play an essential role in regulating the inflammatory reaction of microglia-derived EVs on OPC (Lombardi et al., 2019). Typically, transmitting EVs between astrocytes and microglia can form an inflammatory positive feedback loop, leading to dysregulation and amplification of the neuroinflammatory response.

## Role of Glia-Derived Extracellular Vesicles in Neurodegenerative Diseases

In recent years, glia-derived EVs have been investigated as a potential transmitter of information between neurons and glia, which involves the pathological processes of ND (Table 1). However, the role of glia-derived EVs in the process of ND is complicated. Evidence suggests that activated glial cells accelerate the clearance of pathological proteins by releasing the EVs and thus inhibit the spreading of pathological seeds from cell to cell. On the contrary, the results of more recent studies suggest that glia-derived EVs facilitate the spreading of pathological proteins and promote neurodegeneration (Peng et al., 2020).

**TABLE 1:** Biological properties and effects of glia-derived EVs on neurodegenerative diseases.

Disease	Source of EVs	Stimulation/Culture condition	Main components	Effects of EVs	Markers of EVs	References
AD	Microglia	Aβ1-42	Aβ neurotoxic species	Neurotoxicity	IB4	Joshi et al., 2014
AD	Microglia	Aβ and tau protein	Tau	Accelerating the spread of tau pathology	CD9	Clayton et al., 2021
AD	Astrocyte	Aβ protofibrils	N-terminally truncated Aβ	Neuronal apoptosis and Aβ spreading	Flotillin-1	Söllvander et al., 2016
AD	Astrocyte	Aβ protofibrils	apoE	Intercellular transfer of Aβ pathology by apoE	Flotillin-1, TSG101 and CD9	Nikitidou et al., 2017
AD	Astrocyte	Aβ	Tau/p-tau	Accelerating the spread of tau pathology and forming pre-tangles and NFTs	NA	Chiarini et al., 2017
PD	BV2	Aggregative α-synuclein	MHC-II, TNF-α	Activation of inflammatory response and leading to the neuronal death	CD63	Chang et al., 2013
PD	BV2	Plasma EVs derived from PD patients	Human α-synuclein	Phosphorylated α-synuclein is spread to multiple brain regions of the mouse model	TSG101	Xia et al., 2019
PD	Microglia	Human α-synuclein preformed fibrils	α-synuclein	Promoting the dissemination of α-synuclein in the brain and increasing α-synuclein aggregation in neurons	Tsg101, Alix and CD6	Guo et al., 2020
PD	Microglia	Monomeric α-synuclein	NA	Mitochondrial fission and reducing neurotoxicity	Alix, CD9 and TSG 101	Li et al., 2019
PD	Astrocytes	MPP+	Downregulation of miR-200a-3p	Reducing neuronal apoptosis.	Flotillin-1	Shakespear et al., 2020
PD	Astrocytes	LPS	Upregulation of miR34a	Heightening the vulnerability of DAergic neurons to the neurotoxin	β1 integrin, ribophorin, CD63 and HSP70	Mao et al., 2015
ALS	Astrocytes	C9orf72 mutation	Downregulation of miR-494-3p	Neurite/axonal shortening and neuronal degeneration	NA	Varcianna et al., 2019
ALS	Astrocytes	Overexpressing SOD1 in astrocyte	SOD-1	Inducing selective motor neuron death	Flotillin-1	Basso et al., 2013
ALS	Astrocytes	–	IL-6	Regulation of neuroinflammatory reaction	CD81	Chen et al., 2019

*AD, Alzheimer’s disease; PD, Parkinson’s disease; ALS, amyotrophic lateral sclerosis; NA, not analysis. EVs, extracellular vesicles; Aβ, amyloid-β; apoE, apolipoprotein E; TSG101, Tumor susceptibility gene 101; NFTs, Neurofibrillary Tangles; SOD1, Cu/Zn superoxide dismutase 1.*

### Glia-Derived Extracellular Vesicles in Alzheimer’s Disease

Alzheimer’s disease is characterized by the extracellular plaque deposits of the Aβ and the neurofibrillary tangles of the microtubule-binding protein tau, which activates microglia, induces neuroinflammation, and drives neurodegeneration (DeTure and Dickson, 2019). Glia-derived EVs are now recognized as important mediators in the pathogenesis of AD. Many studies have shown that EVs derived from Aβ or aggregated tau stimulated glia are involved in Aβ aggregation and propagation of tau pathology. When fluorescently labeled Aβ protofibrils were added to a co-culture system of primary neurons and glia, astrocytes could rapidly engulf and store large amounts of Aβ protofibrils. However, no labeled Aβ was found in neurons. The incomplete digestion of Aβ leads to severe endosomal/lysosomal dysfunction in the astrocytes. Eventually, this high intracellular load of toxic results in the secretion of astrocyte-derived EVs containing N-terminally truncated Aβ and the apoptosis of neighboring neurons (Söllvander et al., 2016). A study further analyzed the protein content of EVs released from the neurons and glia co-culture system and found a three-fold increase of apolipoprotein E (apoE) in EVs from Aβ-protofibril-exposed cells than those from unexposed cells. More particularly, these apoE contained EVs are primarily derived from the astrocytes in the co-culture system (Nikitidou et al., 2017).

In addition to the astrocytes, evidence also indicates that microglia can convert aggregated Aβ into neurotoxic forms through the shedding of EVs (Joshi et al., 2014). It has been shown that soluble pre-fibrillar Aβ species are far more toxic than the insoluble fibrils species. After Aβ internalization into microglia, neurotoxic Aβ can be trafficked into EVs. Then EVs’ lipids promote soluble Aβ species from extracellular insoluble aggregates (Joshi et al., 2014). A recent study found that Aβ-associated microglia hyper-secrete EVs to extracellular regions in a humanized APP mouse model, and the expression level of Itgax, a well-established integrin that forms a complex with Integrin beta2 as inactivated-C3b receptor 4, is increased explicitly in the EVs secreted from the microglia, suggesting the contribution of neurodegeneration-induced microglia EVs secretion and the extracellular deposits of Aβ in the pathogenesis of AD (Muraoka et al., 2021).

There is evidence indicating that tau spreading depends on the direct transmission of EVs between neurons and glia. A study *in vivo* used nSMase2- deficient 5XFAD mice with reduced ceramide generation to assess AD-related pathology and demonstrated the release of astrocyte-derived EVs containing tau phosphorylation and Aβ42 plaque burden was increased in the AD mouse model (Dinkins et al., 2016). But contrary to that, evidence from organotypic hippocampal slices shows that neurons and microglia assimilate tau-containing EVs, but not astrocytes (Wang et al., 2017). Ruan et al. have reported that depleting microglia suppresses tau propagation and reduces neuron excitability in the dentate gyrus *in vivo*. Meanwhile, inhibiting the synthesis of microglia-derived EVs reduced tau propagation (Asai et al., 2015; Ruan et al., 2020). Then this team’s most recent study utilized a mouse model of humanized APP mutant knock-in homozygote and found that the activated microglia surrounding the plaque can secrete more EVs that carry more phosphorylated tau (p-tau), comparing to resting microglia. Neurons absorb p-tau contained in EVs and trigger abnormal aggregation of tau protein. Moreover, tau propagation slows down-regulated when microglia are consumed, which is consistent with their previous findings (Clayton et al., 2021).

Microglia-derived EVs have been found to participate in the neuroinflammation in AD. There is evidence that neurons with APP695 mutant can significantly enhance the expression of inflammatory markers, along with higher APP and Aβ1-40 production. Then under the APP695 mutant neurons-microglia co-culture condition, microglia exerted a clearance effect on extracellular APP and Aβ accumulation, probably through internalizing EVs released from neurons in the early stage. However, microglia gradually lose such clearance property and express both pro-inflammatory (iNOS, IL-1β, TNF-α, MHC class II, IL-6) and pro-resolving genes (IL-10 and Arginase 1) in the final stage (Fernandes et al., 2018).

### Glia-Derived Extracellular Vesicles in Parkinson’s Disease

There is considerable evidence supporting that abnormal glia-neuron crosstalk contributes to the progressive death of dopaminergic (DAergic) neurons in the substantia nigra (SN) and the aggregation of α-synuclein in the SN and other areas in the brain and peripheral tissues in PD patients (Meade et al., 2019). α-synuclein released from neurons can be taken up by glia through endocytosis and form inclusion bodies (Tremblay et al., 2019; Marchetti et al., 2020). The glia-derived EVs may exist in distinct phenotypes by carrying various molecules to the DAergic neurons, resulting in neuronal death or neuroprotective functions.

Under inflammatory or neurotoxic conditions, glia generally turns into a harmful phenotype accompanied by the release of EVs, contributing to the increase of DAergic neuron vulnerability and the exacerbation of neuronal death (Wang et al., 2021). Results from the α-synuclein overexpression mouse model indicate that the abnormity accumulated α-synuclein can alter the pro-inflammatory gene expression profile in the glial. What’s more, the increased levels of pro-inflammatory cytokines were associated with the extent of glial accumulation of α-synuclein (Lee et al., 2010). The EVs derived from microglia exposed to aggregated α-synuclein lead to more severe neurotoxicity (Li et al., 2019). When microglia-derived EVs were combined with pro-inflammatory cytokines, the aggregation of α-synuclein would be more significant, suggesting that the internalization of microglia-derived EVs in neurons was accompanied by an immunologically synergic effect, resulting in enhanced neurotoxicity (Guo et al., 2020).

Besides, a growing number of studies focus on microglia-derived EVs as a mediator of α-synuclein transmission in the pathology of PD. EVs-contained α-synuclein oligomers are more easily absorbed by recipient cells and induce more toxicity than the free α-synuclein oligomers outside the cell (Danzer et al., 2012). A recent study reported that when treated with human α-synuclein preformed fibrils, microglia-derived EVs containing α-synuclein contribute to the protein aggregation in the neurons (Guo et al., 2020). What’s more, by stereotaxic injection of α-synuclein preformed fibrils treated microglia-derived EVs into the mouse striatum, phosphorylated α-synuclein can be found in multiple brain regions of the mouse model, including cortex, hippocampus, cerebellum, and SN (Xia et al., 2019).

On the other side, glial have also been discovered to affect neuroprotection against the degeneration of DAergic neurons by releasing specific EVs. For example, astrocytes-derived EVs are shown to reduce MPP+-induced cell death in primary DAergic neuron cultures, with the decreased level of mitogen-activated protein kinase kinase 4, a vital kinase in the c-JNK cell death pathway (Shakespear et al., 2020). Under the stimulation of LPS, astrocytes-derived EVs could heighten the vulnerability of DAergic neurons to neurotoxin by up-regulating the expression of miR-34a, which was shown to targeting anti-apoptotic protein Bcl-2 in DAergic neurons. Whereas inhibition of miR-34a in astrocytes-derived EVs can postpone DAergic neuron loss under the inflammatory stimuli (Mao et al., 2015).

### Glia-Derived Extracellular Vesicles in Amyotrophic Lateral Sclerosis

Amyotrophic lateral sclerosis is a progressive ND characterized by motor neuron degeneration and death, with gliosis replacing lost neurons (Zhang et al., 2014). Although the mechanisms underlying ALS remain unclear, many pathologic processes have been implicated, such as protein aggregation, impaired protein degradation, and neuroinflammation (Suk and Rousseaux, 2020). The mutual effect between motor neurons and glia is essential in the outcome of ALS. Cu/Zn superoxide dismutase 1 (SOD1) was the first gene associated with ALS, accounting for about 10–20% of familial ALS. It has been shown that mutant SOD1 overexpression primary astrocytes can release mutant SOD1-containing EVs, leading to selective motor neuron death (Basso et al., 2013). Studies in animal models also indicate that astrocyte-derived EVs contribute to the spreading of pathogenic SOD1 (Silverman et al., 2019). Astrocyte-derived EVs from brains and spinal cords of the SOD1-G93A ALS mouse model and the spinal cord of ALS patients with SOD1 mutant have been found to contain abundant misfolded and non-native disulfide-cross-linked aggregated SOD1 (Silverman et al., 2019). In addition, human-induced astrocytes from ALS patients carrying C9orf72 mutations were used to explore the role of astrocyte-derived EVs in the neurotoxicity of ALS. miRNA cargo is altered in the astrocyte-derived EVs of C9orf72-ALS patients, contributing to dysregulation of neurite network maintenance and motor neuron survival (Varcianna et al., 2019). As neuroinflammation participates in the pathogenesis of ALS, a study evaluated the expression level of IL-6 in the astrocyte-derived EVs from the plasma of sporadic ALS patients. And the results showed that the level of IL-6 in astrocyte-derived EVs was increased in ALS patients and positively associated with the disease progression, suggesting the increased inflammatory cascade in the CNS of ALS patients (Chen et al., 2019). The involvement of glia-derived EVs in glia-to-neuron communication in the context of ALS has not been explored in-depth, which deserves further investigation.

## Extracellular Vesicles as Biomarkers for Neurodegenerative Diseases

One of the clinical challenges of ND is the difficulties in making a definitive diagnosis at the early stages and predicting the disease progression. The unmet demand to identify reliable biomarkers for early diagnosis and management of the disease course has attracted much attention (Li and Le, 2020). EVs harbor proteins and nucleic acids that are likely to indicate pathogenic processes occurring in hardly accessible tissues, such as the CNS, for their potential of tracking down the donor cell. Therefore, peripheral EVs may hold promise for biomarker discovery in ND.

Peripheral glia-derived EVs were shown to be enriched in the plasma and cerebrospinal fluid (CSF) of AD patients with similar characteristics to those derived from the CNS, such as containing glia-specific cargo proteins or miRNAs, which involves the cellular transmission of AD-related pathology. For instance, plasma astrocyte-derived EVs levels of BACE-1, γ-secretase, soluble Aβ42, soluble APPβ, P-T181-tau, and P-S396-tau were markedly increased compared with those in neuron-derived EVs. What’s more, levels of BACE-1 and soluble APPβ were significantly higher in astrocyte-derived EVs of AD patients than in those of healthy controls (Goetzl et al., 2016). Goetzl et al. have found that astrocyte-derived EVs from AD patients contain high levels of multiple complement components (C1q, C4b, C3b, Bb, C3d, factor B, and factor D), which is known to contribute to the formation of the membrane attack complex (MAC) (Goetzl et al., 2018). Besides, Muraoka et al. (2020a) performed the first proteomic profiling of EVs isolated from post mortem AD brain tissues and found high glia-specific factors in the CNS- derived EVs from AD patients. ANXA5, VGF, GPM6A, and ACTZ were identified by machine learning quantitative proteomics datasets, which can distinguish AD and control CNS-derived EVs with 88% accuracy. Another recent study also examined the proteomic profiling of the EVs separated from the CSF of AD, cognitive impairment (MCI) patients, and healthy controls. The results indicated that astrocyte-specific molecules were enriched in AD compared to MCI, and HSPA1A, NPEPPS, and PTGFRN can be applied to monitor the progression of MCI to AD (Muraoka et al., 2020b).

It has been reported that the level of CNS-derived EVs contained α-synuclein is significantly higher in the plasma of PD patients, which may serve as a potential biomarker for PD (Shi et al., 2014; Si et al., 2019). A longitudinal study reported that α-synuclein level in the plasma EVs is significantly higher in patients with early stage PD than healthy controls, and the increased α-synuclein is associated with a higher risk for motor symptom progression in PD (Niu et al., 2020). It has to be taken into account that the outcome depends on the donor cells of the EVs carrying α-synuclein in the plasma. However, so far, no data relevant to α-synuclein in specific glia-derived EVs as potential biomarkers for PD has been reported. A recent study evaluated the plasma levels of neuron-derived, astrocyte-derived, and oligodendrocyte-derived EVs in patients with PD, multiple system atrophy (MSA), progressive supranuclear palsy. The neuron-derived EVs showed a significant increase in PD compared to control and MSA. And the plasma levels of oligodendrocyte-derived EVs and the ratio of oligodendrocyte/neuron-derived EVs showed a substantial correlation with UPDRS part III scores in the patients with MSA (*r*^2^ = 0.57) and PD (*r*^2^ = 0.51), respectively (Ohmichi et al., 2019). In addition, alterations of other proteins, nucleic acids, and miRNAs have also been detected in the peripheral EVs from PD patients (Fraser et al., 2016; Zhao et al., 2019). Proteomic analysis of urinary EVs has shown that the combination of SNAP23 and calbindin reached the diagnostic performance of PD with 77% sensitivity and 85% specificity (Wang et al., 2019). It has been shown that circulating EVs have altered levels of miR-195, miR-24, let-7-c-3p, miR-331-5p, and miR-505 in PD patients compared with controls (Cao et al., 2017; Yao et al., 2018). Nevertheless, little was known about the peripheral EVs, carrying a cargo of protein or miRNAs as potential biomarkers derived from what kind of donor cells (cells in the blood, neurons, or glia). Within this context, more efforts in identifying the specific EV-donor cell are needed to clarify the role of glia-derived EVs in the diagnosis and prognosis of PD.

Plasma-derived EVs have also been analyzed recently in human samples as well as in animal models by Pasetto et al. (2021). EVs from ALS patients and two ALS-related mouse models show a distinct size distribution with smaller mean diameters and lower amount of Hsp90 than that of controls (Pasetto et al., 2021). In this study, the level of cyclophilin A in EVs, together with EV size distribution show potentials in identifying patients with fast or slow disease progression (Pasetto et al., 2021).

## Extracellular Vesicles for Therapy of Neurodegenerative Diseases

Efforts to study EVs for therapeutic applications in diseases are rapidly increasing. EVs bear the great advantage of being stable in the blood, the ability to overcome the BBB, and possessing specific cell-targeting capabilities, therefore delivering their cargoes within the CNS (Aryani and Denecke, 2016). Therapeutic EVs usually comprise diverse cargoes include RNA, proteins, and drugs. The key strategic issues that need to be addressed are the choice of therapeutic cargoes, promoting EV stability, tissue targeting, and functional cargo delivery to recipient cells (György et al., 2015). The identification of EVs as tools for delivering cargo molecules to diseased tissues makes these circulating shuttles possible targets for therapeutic development.

Accumulating evidence of preclinical therapeutic efficacy indicates that EVs could be a potential regenerative substance in the treatment of ND. Several types of stem cells-derived EVs have been used for nerve regeneration as EV donors in preclinical trials through induction of regenerative phenotypes, immune regulation, and apoptosis inhibition (Yin et al., 2020; Lee and Kim, 2021). Mesenchymal stem cells (MSCs)-derived EVs could promote neurogenesis and endogenous angiogenesis and reduce the neuroinflammation in the brain injury model (Zhang et al., 2015). It was shown that adipose MSCs-derived EVs contain neprilysin, an enzyme that degrades Aβ, which effectively reduces intracellular and secreted Aβ levels, suggesting a possible treatment application for AD (Katsuda et al., 2013; De Godoy et al., 2018). A study using a 3D culture model of stem cells derived from the dental pulp of human exfoliated deciduous teeth (SHEDs) and DAergic neurons and found that EVs derived from SHEDs rescued DAergic neurons from 6-hydroxy-dopamine (6-OHDA)-induced apoptosis and may be considered as a potential therapeutic tool in the treatment of PD (Jarmalavičiūtė et al., 2015). In addition to *in vitro*, intranasal administration of EVs derived from SHEDs also presented the improvements in motor function and normalization of tyrosine hydroxylase (TH) expression in the SN of 6-OHDA treated PD rat model (Narbute et al., 2019). Lombardi et al. (2019) evaluated the effects of EVs released from either pro-inflammatory or pro-regenerative microglia on the demyelinated lesions of OPCs, and the results showed that pro-inflammatory microglia-derived EVs blocked remyelination, whereas EVs released by microglia co-cultured with immunosuppressive MSCs accelerated OPC recruitment and myelin repair.

Since the beneficial effects of glia-derived EVs and their potential therapeutic actions on ND, it would be crucial to engineering EVs with a beneficial role. Many studies have been conducted to engineer EVs to express beneficial biomolecules such as proteins, mRNA, and miRNAs by using various donor cells to treat ND. The therapeutic potential of EVs-mediated interfering RNA (siRNA) delivery was firstly demonstrated by the knockdown of BACE1, a therapeutic target of AD, in EVs derived from autologous dendritic cells. After intravenous injection, EVs are explicitly delivered to neurons, microglia, oligodendrocytes in the brain, resulting in the specific BACE1 gene knockdown (Alvarez-Erviti et al., 2011). Haney et al. (2013) revealed that EVs contained catalase genetic material, active catalase, and NF-κb, which were released from the transfected macrophages, can efficiently transfer their contents to contiguous neurons resulting in a reduction of inflammation and neuroprotection in a mouse model of PD. The following administered to the PD mice macrophages overexpressing glial-derived GDNF, again with the analog results of improving neuroinflammation and neurodegeneration (Zhao et al., 2014).

Given two opposite effects of glia-derived EVs in ND, treatment strategies utilizing EVs may bring a wrong side at the same time. Therefore, some studies focused on inhibiting glia-derived EVs secretion from diminishing the propagation of pathological protein in ND. For instance, ceramide-enriched EVs have been shown to exacerbate AD-related brain pathology by promoting the aggregation of Aβ. A study reported that reducing the secretion of astrocyte-derived EVs by nSMase2 improves pathology and cognition in an AD mouse model (Dinkins et al., 2016). Besides, it has been shown that blocking dynamin-related protein 1 can reduce the release of EVs and spread of α-synuclein pathology from neurons to neurons and from microglia to neurons, which plays a role in neuroprotection through both mitochondrial and autophagy-lysosomal pathways (Fan et al., 2019). MSCs-derived EVs were shown to modulate the detrimental effect of glia in neurodegenerative conditions. A study found that the intranasal route of administration of cytokine-preconditioned MSCs-derived EVs contributes to the immunomodulation and neuroprotection in an AD transgenic mouse model. MSC-EVs can deliver into the brain, thereby increasing the dendritic spine density and depressing microglia activation (Losurdo et al., 2020). Furthermore, it was shown that MSCs-derived EVs could effectively trigger the microglia polarization from M1 to an M2 phenotype and improve the survival of neurons through down-regulation of the pro-inflammatory cytokines (Sun et al., 2018).

Although many studies in the mouse model have shown promising results of the application of EVs in the treatment of ND, crucial species differences in the function of the BBB and targets of EVs to the brain may exist between humans and mice. Little research so far has dealt with the effect of EVs on the human brain. Overall, it will be of great value to clarify the glia-derived EVs content and distinct functional impacts on ND and improve the techniques of EVs engineering. These efforts will open up a new field, implementing the characterization of glial as a potential producer of a novel generation of ND nanotherapeutics.

## Conclusion and Future Perspectives

**FIGURE 1:**
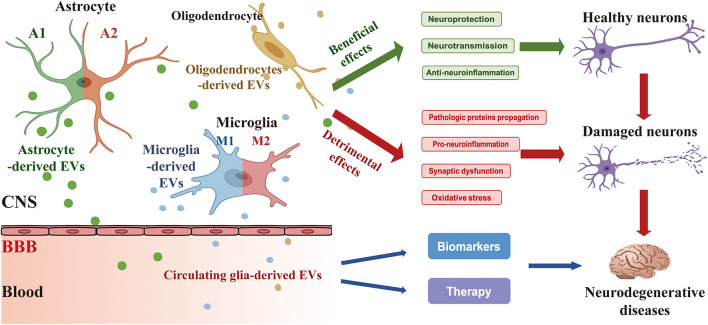
Secretion of EVs derived from glia (astrocyte, microglia, and oligodendrocyte) and their effects on neurons and ND. Glia-derived EVs have a beneficial and/or detrimental impact on the process of ND like a “double-edged sword.” Under condition normal circumstances, glia-derived EVs contribute to neuroprotection, neurotransmission, and anti-neuroinflammation. However, in response to the different stimuli such as toxic aggregated proteins and neuroinflammation, glia-derived EVs modify their cargo content and have detrimental effects on pathologic proteins propagation, pro-neuroinflammation, synaptic dysfunction, and oxidative stress. In addition, circulating glia-derived EVs hold considerable potential for clinical applications in the diagnosis and treatment of ND. CNS, central nervous system; BBB, blood-brain barrier.

Glia is abundant in the CNS and contributes to supporting the essential functions of neurons, including synapse formation and plasticity, neurotransmitter buffering, secretion of neuroactive agents, and modulation of neuroinflammatory reactions. The crucial role of glia-derived EVs in glia-to-neuron communications has been widely noted in recent years. Under normal circumstances, glia-derived EVs play an important part in maintaining the normal neuronal function, promoting neurite outgrowth and neuronal survival. However, in response to the different stimuli such as toxic aggregated proteins and neuroinflammation, glia-derived EVs modify their cargo content and have dual effects on the survival of the neurons like a “double-edged sword.” In addition, as microglia are heterogeneous with diverse phenotypes of pro-inflammatory M1 and anti-inflammatory M2 phenotypes, EVs from M1 or M2 phenotypes of microglia also present various functional properties triggered by different stimuli (Figure 1).

In recent years, glia-derived EVs have been shown to participate in the pathological processes of ND. It is worth noting that the role of glia-derived EVs in the ND also results in totally different outcomes. On the one hand, the glial can promote clearance and inhibit the spreading of pathological proteins by releasing the EVs. On the other hand, glia-derived EVs have been shown to facilitate the propagation of pathological proteins and ultimately result in neurodegeneration. The sources of heterogeneity may be attributable to the differential expression of the main components in the glia-derived EVs.

Glia-derived EVs hold considerable potential for various clinical applications. EVs can overcome the BBB, be stable in the blood, and have the possibility of tracking down the donor cell, therefore delivering their cargoes within the CNS. For this reason, various studies have been conducted in discovering potential biomarkers and therapeutic targets for ND by focusing on the CNS-derived EVs. However, investigations on the glia-specific EVs in identifying biomarkers for ND are rare, which may be due to the mix of different EVs released from various donor cells that exist in the peripheral circulation. Approaches to label different cell-specific EVs are required in future studies on glia-derived EVs *in vivo*. Moreover, concerning the beneficial and detrimental roles of the glia-derived EVs in the pathogenesis of ND, further investigations of the specific associated biological molecules in the glia-derived EVs are necessary. Based on more understanding of the core molecule expressed in the glia-derived EVs, engineering EVs to express beneficial biomolecules will provide a novel and efficient tool for the therapeutic applications on ND.

## Author Contributions

WL, TL, and SL designed the project of this manuscript. TL, XT, SL, MA-N, and WL contributed to the drafting of the manuscript. TL, MA-N, and WL revised the manuscript. All the authors edited and approved the final version of the manuscript.

## Conflict of Interest

The authors declare that the research was conducted in the absence of any commercial or financial relationships that could be construed as a potential conflict of interest.

## Publisher’s Note

All claims expressed in this article are solely those of the authors and do not necessarily represent those of their affiliated organizations, or those of the publisher, the editors and the reviewers. Any product that may be evaluated in this article, or claim that may be made by its manufacturer, is not guaranteed or endorsed by the publisher.
